# Evaluation of a multifaceted implementation strategy for semi-automated surveillance of surgical site infections after total hip or knee arthroplasty: a multicentre pilot study in the Netherlands

**DOI:** 10.1186/s13756-024-01418-0

**Published:** 2024-06-13

**Authors:** Manon Brekelmans, Titia Hopmans, Maaike van Mourik, Sabine de Greeff, Julie Swillens, Stephanie van Rooden

**Affiliations:** 1 Centre for Infectious Diseases Control, National Institute for Public Health and the Environment, Bilthoven, the Netherlands; 2 Department of Medical Microbiology and Infection Control, University Medical Centre Utrecht, Utrecht, the Netherlands; 3 Scientific Centre for Quality of Healthcare (IQ Healthcare), Radboud Institute for Health Sciences (RIHS), Radboud University Medical Centre, Nijmegen, The Netherlands

**Keywords:** Automated surveillance, Implementation science, Surgical site infections

## Abstract

**Introduction:**

To promote the nation-wide implementation of semi-automated surveillance (AS) of surgical site infection after hip and knee arthroplasty, the Dutch National Institute for Public Health and the Environment (RIVM) deployed a decentralised multifaceted implementation strategy. This strategy consisted of a protocol specifying minimum requirements for an AS system, supported by a user manual, education module, individual guidance for hospitals and user-group meetings. This study describes an effect evaluation and process evaluation of the implementation strategy for AS in five frontrunner hospitals.

**Methods:**

To evaluate the effect of the implementation strategy, the achieved phase of implementation was determined in each frontrunner hospital at the end of the study period. The process evaluation consisted of (1) an evaluation of the feasibility of strategy elements, (2) an evaluation of barriers and facilitators for implementation and (3) an evaluation of the workload for implementation. Interviews were performed as a basis for a subsequent survey quantifying the results regarding the feasibility as well as barriers and facilitators. Workload was self-monitored per profession. Qualitative data were analysed using a framework analysis, whereas quantitative data were analysed descriptively.

**Results:**

One hospital finished the complete implementation process in 240 person-hours. Overall, the elements of the implementation strategy were often used, positively received and overall, the strategy was rated effective and feasible. During the implementation process, participants perceived the relative advantage of AS and had sufficient knowledge about AS. However, barriers regarding complexity of AS data extraction, data-infrastructure, and validation, lack of capacity and motivation at the IT department, and difficulties with assigning roles and responsibilities were experienced.

**Conclusion:**

A decentralised multifaceted implementation strategy is suitable for the implementation of AS in hospitals. Effective local project management, including clear project leadership and ownership, obtaining commitment of higher management levels, active involvement of stakeholders, and appropriate allocation of roles and responsibilities is important for successful implementation and should be facilitated by the implementation strategy. Sufficient knowledge about AS, its requirements and the implementation process should be available among stakeholders by e.g. an education module. Furthermore, exchange of knowledge and experiences between hospitals should be encouraged in user-group meetings.

**Supplementary Information:**

The online version contains supplementary material available at 10.1186/s13756-024-01418-0.

## Introduction

Surveillance of healthcare associated infections (HAI) is a cornerstone of infection prevention programs. Surveillance networks focusing on HAI aim to provide insight in the presence and trends of HAI within and between hospitals or countries. These networks subsequently identify risk factors and generate benchmark data to target interventions to improve quality of care [1]. HAI surveillance is mainly performed by manual chart review, generally by infection control practitioners (ICP). However, this method is very time-consuming and labour-intensive [2, 3]. Moreover, manual surveillance is subjective to interpretation differences and the quality of the results of the surveillance is effort dependent [4–6].

To address these deficiencies, (semi-)automated surveillance (AS) of HAI could replace manual surveillance [7, 8]. AS is defined as any form of surveillance where manual decisions are (partially) replaced by an automated process, utilizing routine care data from electronic health records (EHR) [7]. A semi-automated surveillance system consists of (1) automated selection of the patients/procedures that should be included in the surveillance, (2) extraction of source data from the EHR and (3) application of an algorithm to classify patients/procedures into high or low probability of an infection according to the case-definition. Thereafter, infections are manually confirmed and registered for patients/procedures that were assigned a high probability of infection. Patients/procedures with low probability are directly registered as ‘no infection’ [9]. In addition, additional variables for, among other things, risk adjustment, are automatically extracted from the EHR. Currently, AS is mainly being applied in research settings and individual hospitals rather than on a large scale in surveillance networks [8]. Central coordination in such networks is important to ensure sound and uniform methodology of AS across hospitals as a basis for comparable and reliable results, just as with manual surveillance.

To facilitate development of AS on a large scale, the PRAISE roadmap (Providing a Roadmap of Automated Infection Surveillance in Europe) has been developed in 2021 [7]. Here, a lack of evidence of effective implementation strategies for the large-scale implementation of AS was underlined. This roadmap served as a base to develop a decentralised multifaceted implementation strategy for AS developed by the Dutch national surveillance network for healthcare associated infections (PREZIES), a collaboration of the National Institute of Public Health and the Environment (RIVM) and participating hospitals. The implementation strategy aimed to implement an semi-automated surveillance system for surgical site infections (SSI) after total hip arthroplasty (THA) or knee arthroplasty (KA) [10]. These high-volume procedures are included in many surveillance systems [11, 12] and have a low incidence of SSI. In addition, clinical procedures in cases of a (suspected) SSI are relatively well standardized. A classification algorithm for deep SSI after THA or KA has been developed and validated in Dutch hospitals previously, showing a sensitivity for deep SSI of 93–100%, and a ~ 95% reduction of the number of surgeries requiring manual assessments [13, 14]. All this together, makes this a surveillance target with substantial gains of AS and feasible to implement.

In this study, we aimed to perform an effect evaluation and process evaluation of the implementation strategy parallel to the start of implementation of a semi-automated surveillance module for SSI in five frontrunner hospitals in The Netherlands. Results of this study can contribute to further optimize the implementation of AS on a large scale.

## Methods

### Multifaceted implementation strategy

To coordinate large-scale implementation of AS of SSI after THA or KA, the RIVM as coordinating centre developed a multifaceted implementation strategy, which was based on the PRAISE roadmap [7], previous experiences with implementation projects of AS systems [15, 16], expert meetings, advisory boards and data experts [17]. Given the large variety of EHR system used in Dutch hospitals, AS systems are locally designed according to a standard protocol. AS was implemented initially in five so-called frontrunner hospitals, before national roll-out. Selection of these frontrunner hospitals was based on readiness assessment, identification of early adopters and formal commitment by hospital management. The implementation strategy consisted of five elements: a protocol including minimal requirements of an AS system [10], a user manual [10], an education module, individual guidance by the coordinating centre and user-group meetings (Table 1 A). Although no formal barrier and facilitator assessment was performed before the development of the implementation strategy, recommendations for successful implementation of AS were provided based on previous projects [15], individual expert consultation and expert meetings. Recommendations included obtaining commitment of all stakeholders before the start of the implementation, development of a project plan, emphasis of the importance of clear communication between stakeholders and on algorithm selection, development and validation. Those were addressed in the implementation strategy elements.

**Table 1:** Specification of implementation strategy elements [18] (**A**) and evaluation of their feasibility (**B**)

**A**		**Protocol including minimal requirements [** 10 **]**	**User manual [** 10 **]**	**Education module**	**Guidance by the coordinating centre**	**User-group meetings**
	Content	Definitions (surveillance population and case) Acceptance criteria algorithm Data specifications Minimal requirements AS system Internal and external validation	How to write local implementation plan Formation of a project group Involvement of stakeholders of the implementation of AS Identification and solving local barriers for implementation	Basic principles of AS Validation of source data and algorithm components Hospital information systems Information standards Governance Development of AS system Maintenance	Discuss progress Questions or issues	Exchange experiences and examples between hospitals Updates/information from the coordinating centre
	Developed by (actor)	Coordinating centre Expert and stakeholder consultation	Coordinating centre	Coordinating centre and AS Expert (coordination & education); Experts specific topics (education)	Coordinating centre	Coordinating centre– interactive participative meetings with intended users
	Goal (implementation outcome affected)	Implementation Sustainability	Adoption Implementation Sustainability	Implementation Sustainability	Adoption Implementation Sustainability	Adoption Implementation Sustainability
	Elements ERIC [17]	Develop a formal implementation blueprint Develop and organize quality monitoring systems Promote adaptability	Develop educational materials Develop an implementation glossary	Conduct educational meetings Create a learning collaborative	Audit and provide feedback Conduct local needs assessment Provide ongoing consultation Tailor strategies	Use advisory boards and workgroups Create a learning collaborative Promote network weaving Capture and share local knowledge
	Timing roll-out	Start of the implementation	Start of the implementation	Day 1: 2 months after start Day 2: 5 months after start	Every three months or whenever requested, physical and online meetings	Quarterly meetings 1 h in online format
	Intended users	Project leader Team members	Project leader Team members	Project leader Team members	Project leader Team members	Project leader Team members
	Type of element	N.A.	Passive	Active	Active	Active
**B**	**Results survey**					
	Exposed	N.A.	12/17 (71%)	8/12 (67%)	13/17 (76%)	10/17 (59%)
	Users by profession *n /exposed (%)*	N.A.	8/12 (67%) ICP: 6 IT: 1 Project manager: 1	4/8 (50%) ICP: 4	7/13 (54%) ICP: 5 MM: 1 IPC management: 1	6/10 (60%) ICP: 5 MM: 1
	Users by role^a^ *n/total users (%)*	N.A.	Project leader: 4/8 (50%) Team member: 4/8 (50%) Developer: 5/8 (63%) User: 5/8 (63%) Leader department: 2/8 (25%)	Project leader: 3/4 (75%) Team member: 3/4 (75%) Developer: 3/4 (75%) User: 3/4 (75%) Leader department: 1/4 (25%)	Project leader: 3/7 (43%) Team member: 6/7 (86%) Developer: 4/7 (57%) User: 4/7 (57%) Leader department: 3/7 (43%)	Project leader: 3/6 (50%) Team member: 5/6 (83%) Developer: 4/6 (67%) User: 4/6 (67%) Leader department: 2/6 (33%)
	Phase use^a^ *n/total users (%)*	N.A.	At the start: 5/8 (63%) During implementation: 6/8 (75%) End of implementation: 2/8(25%) Specific questions: 4/8 (50%)	At the start: 3/4 (75%) During implementation: 3/4 (75%) Specific questions: 4/4 (100%)	At the start: 6/7 (46%) During implementation: 3/7 (23%) End of implementation: 1/7 (8%) Specific questions: 4/7 (31%)	N.A.
	Recommendation to colleagues^b^	N.A.	88%	N.A.	100%	67%
	Self-reported effectiveness^c^	N.A.	38%	100%	100%	83%

^*a*^ *Multiple answers possible*

^*b*^ *Recommendation to colleagues percentage of respondents that would recommend the element to colleagues*

^*c*^ *Self-reported effectiveness: percentage of respondents that answered that the element makes implementation easier*

*ICP: infection control practitioner; IPC: infection prevention and control; IT: information technology or business intelligence specialist; MM: medical microbiologist; N.A.: not applicable*

### Automated surveillance system

**Fig. 1:**
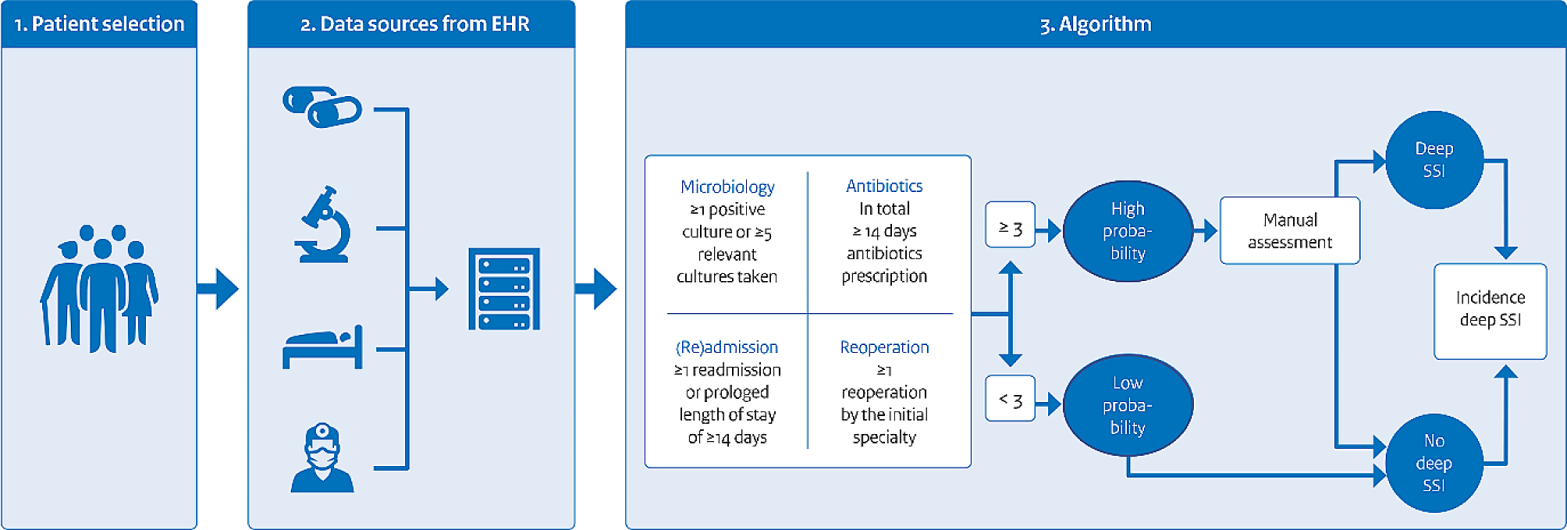
Design of an automated surveillance system for surgical site infections after hip or knee arthroplasty. EHR: electronic health record; SSI: surgical site infection; figure adapted from PREZIES [10]

Central to the automated surveillance system for deep SSI after THA or KA (Fig. 1) is the classification algorithm developed by Sips et al. [14]. In short, patients who underwent primary THA or KA procedures are selected for surveillance based on operating records. Subsequent to data collection from different data sources and application of the algorithm, procedures classified as ‘high probability of SSI’ undergo manual assessment to confirm deep SSI. Due to differences in EHR systems between hospitals, not all source data may be available in every hospital or local procedures may deviate from those specified in the protocol. Hence, this protocol allows for motivated deviations of the algorithm as long as performance falls within pre-specified acceptance criteria.

### Study design

Parallel to the deployment of the multifaceted implementation strategy, starting in April 2022, an effect evaluation and process evaluation of the implementation strategy of AS were performed until June 2023 (the end of the study period) in the five frontrunner hospitals as a pilot for national implementation (Table 2). The implementation strategy is described following the recommendations for specifying and reporting of implementation strategies [18], the consolidated criteria for reporting qualitative studies (COREQ) [19] for the reporting of the qualitative interviews, and the checklist for reporting of survey studies (CROSS) for reporting of the survey [20] (Supplement 3–5). The medical ethical review board NedMec confirmed that the Medical Research Involving Human Subjects Act (WMO) does not apply to this study (reference number 22/753).

**Table 2:** Study design, outcomes and data sources

Evaluation	Outcome	Detailed outcome	Framework	Data source
Effect evaluation	Phase of implementation	Adoption, implementation and sustainment	CFIR outcomes addendum [21]	Documentation of individual guidance by coordinating centre
Process evaluation	Feasibility	Actual use	Hulscher et al. [22]	Attendance lists by coordinating centre Interviews and survey
		Experiences with the implementation strategy Acceptability Demand Implementation Practicality Self-reported effectiveness	Hulscher et al. [22] Bowen et al. [23] Bowen et al. [23] Bowen et al. [23] Bowen et al. [23] Hulscher et al. [22]	Interviews and survey
	Barriers and facilitators	Innovation Outer setting Inner setting Individual characteristics Implementation process	CFIR [24]	Interviews and survey
	Workload for implementation	Person-hours spent on implementation	N.A.	Self-registration by forms

### Effect evaluation

Based on the Consolidated Framework of Implementation Research (CFIR) Outcomes Addendum [21], concretised for development and implementation of an AS system, the effect of the implementation strategy was determined for each frontrunner hospital at the end of the study period in terms of achieved implementation phase. The three phases include adoption (i.e. internal commitment to start the implementation project), implementation (i.e. completion of internal validation of an operating AS system) and sustainment (i.e. successful external validation of AS system and processes for maintenance and sustainability) [21, 25, 26]. Based on these three pre-defined implementation phases, the progress of the implementation was determined and documented during the individual guidance by the coordinating centre.

### Process evaluation

To evaluate the implementation strategy and to identify points for improvement, we studied the feasibility, barriers and facilitators as well as workload. Feasibility of the implementation strategy was assessed following the concepts of Hulscher et al. [22], being actual exposure to, and use of the implementation strategy, and experiences of those exposed to the implementation strategy. The experiences are specified using the framework of Bowen et al. [23] and we selected concepts that were applicable to this pilot study: (1) acceptability (i.e. satisfaction with content of elements, points for improvement, recommendation to colleagues), (2) demand (i.e. desire to be exposed, reasons (not) to use elements, phase of use), (3) implementation (i.e. satisfaction with way of exposure) and (4) practicality (i.e. usefulness of parts within element, moments of use/usefulness) of the implementation strategy elements. Furthermore, self-reported effectiveness (i.e. to which extent the element has affected implementation success) of the implementation strategy elements was studied [22] (Table 2, Supplement 1)). Barriers and facilitators for implementation of AS were identified by means of the following domains of the CFIR framework [24]: innovation, outer setting, inner setting, individuals, and implementation process. This framework is widely used and has been updated in 2022 based on user feedback. These frameworks formed the structure and content of data collection and data analysis. Furthermore, workload for implementation was assessed and expressed in person-hours spent. We aimed to include the complete spectrum of stakeholders involved in local implementation, being ICP, medical microbiologists (MM), information technology or business intelligence (IT) specialists, orthopaedic surgeons, project managers and management staff, representing all participating hospitals.

### Data collection

Interviews were conducted to explore the feasibility of the strategy elements and barriers and facilitators for implementation of AS. The interview guide (Supplement 2) covered participant characteristics, feasibility of the implementation strategy elements [22, 23] and barriers and facilitators for implementation [27] and was tested within the research team. The interview guide was adapted to the profession of the participant. Stakeholders were invited for the interviews by the researcher (MB) by e-mail and written informed consent was obtained. The interviews were conducted by a student, initially supervised by the researcher (MB), and lasted for approximately 30 min. The interviews were recorded and transcribed verbatim for framework analysis [23, 24].

The results of the interviews served as a base for an online survey, complemented by additional concepts of CFIR [24] to quantify the results in order to assess the relative importance of feasibility aspects, barriers and facilitators, to eventually improve the implementation strategy [22]. The survey consisted of 3 parts: (1) participant characteristics including age group, sex, profession and working experience, (2) feasibility of the implementation strategy elements and (3) barriers and facilitators for implementation on a 5-point Likert scale, ranging from strongly disagree to strongly agree. The participants were asked to identify themselves with one or more roles during the implementation, including project leader, project team member, innovation developer, innovation user or leader at a department level [24]. The survey was developed in Formdesk (Innovero Software Solutions B.V.) and tested by the research team and three team members from non-frontrunner hospitals. The survey was distributed approximately one year after the start of implementation with reminders after 2 and 4 weeks. Surveys were anonymous and could not be related to a specific hospital. Written informed consent was obtained.

Additionally, actual use of the education module, guidance by the coordinating centre and user-group meetings was monitored using an attendance list by the coordinating centre for all participants, also including non-respondents of the survey.

Data regarding the workload for implementation was collected in a time registration form provided by the study team. The project leader, team members and AS developers were asked to register their activities of implementation, the person-hours spent on these activities and the date on which these activities were executed.

### Data analysis

The interviews were analysed anonymously. Deductive coding (framework-driven) [28] of the interviews was performed using MAXQDA (VERBI Software, 2021), based on the constructs of the feasibility framework of Bowen et al. [23] and CFIR [24] for barriers and facilitators. Additionally, researchers critically examined whether the results actually fit within the chosen frameworks. Coding was performed by two researchers independently (MB, SG), and when no consensus was reached, a senior researcher (JS) was consulted.

Only fully completed surveys were included in the analysis. For the barriers and facilitators, all propositions with > 2 responses were included in the analysis. Negatively formulated propositions, e.g. “setting up a data-infrastructure was complex”, with (strong) agreement of ≥ 33% of the respondents were classified as a barrier; else it was neither classified as a barrier, nor as a facilitator. If more than 67% of the respondents (strongly) agreed with a proposition positively formulated, e.g. “AS saves time”, then we classified it as a facilitator. When 33–67%, of the respondents (strongly) agreed, then the variable was classified as a barrier or facilitator depending on the context of the interviews. If less than 33% of the respondents (strongly) agreed, then we classified it as a barrier [29].

Descriptive analyses were performed to analyse the survey results, actual use of elements of implementation strategy and workload, using IBM SPSS Statistics V28.0 (IBM Corp, Armonk, New York).

## Results

### Effect evaluation

All hospitals indicated that there was internal commitment to start the implementation of AS (adoption). Only one of the five hospitals managed to complete the entire implementation process within the study period, while the other hospitals were in the implementation phase (*n* = 4).

### Process evaluation

#### Study population

Five interviews with different stakeholders from four hospitals: two ICP, one medical microbiologist, one IT specialist and one project manager served as a base for the survey. A total of 27 surveys were distributed directly to each stakeholder and 17 respondents (63%) completed the survey within the five hospitals (Table 3).

**Table 3:** General characteristics of the study population

	Interviews (*n* = 5)	Survey (*n* = 17)
**Profession**	*n* ** (% of total respondents)**	*n* **/invited (% of total respondents)**
ICP	2 (40)	7/8 (41)
MM	1 (20)	4/5 (24)
IT	1 (20)	2/5 (12)
Orthopaedic surgeon	0 (0)	1/4 (6)
IPC management	0 (0)	2/3 (12)
Project manager	1 (20)	1/2 (6)
Role during implementation project, by profession, n (% of total respondents)^a^	N.A.	
Project leader		4 (24)
ICP		3 (75)
Project manager		1 (25)
Team members		6 (35)
ICP		4 (67)
MM		1 (17)
IPC management		1 (17)
Developers		8 (47)
ICP		4 (50)
MM		2 (25)
IT		2 (25)
Users		6 (35)
ICP		5 (83)
Orthopaedic surgeon		1 (17)
Leader department		6 (35)
ICP		3 (50)
MM		1 (17)
IPC management		2 (33)
Sex – female, *n* (%)	2 (40)	11 (65)
Age, *n* (%)		
18–35 year	N.A.	3 (18)
36–50 year	N.A.	8 (47)
51–68 year	N.A.	6 (35)
Years of working experience, median (IQR)	8 (16)	10 (15)
Years working by employer, median (IQR)	9 (7)	8 (11)

^*a*^*Roles from CFIR* [24], *adapted to the context of this study*

*ICP*: *infection control practitioner; IPC: infection prevention and control; IT: information technology or business intelligence specialist; MM: medical microbiologist*

### Feasibility

Results of the feasibility evaluation of the implementation strategy elements based on survey results are presented in Table 1B.

#### User manual

The user manual was received by 12 out of 17 respondents (71%) and actually used by eight of those 12 (67%) respondents, mainly to write the project plan (6/8). The users were mostly ICP reporting their roles as project leader, developers and/or users. Overall, respondents were neutral in whether the manual facilitated implementation, but would recommend it to colleagues. With a better description of the role and responsibilities of the project leader and more hands-on tips and best practices to increase practicality, the manual could be improved.

#### Education module

**Fig. 2:**
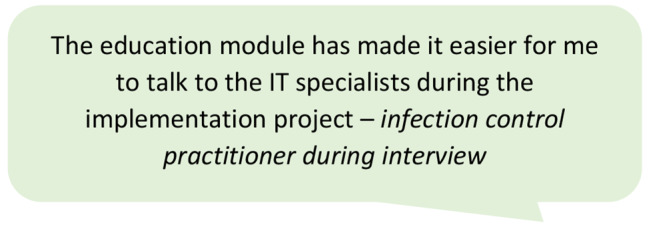
Quote about education module

The education module was attended by eight individuals representing all hospitals: six ICP, one medical microbiologist and one IPC management staff member. Among the survey respondents, eight of 12 (67%) were familiar with this module, of whom four (50%) actually attended. These were all ICP and motivated by a lack of knowledge for the implementation of AS (3/4). The education module facilitated implementation of AS (4/4, Fig. 2) but could be improved by providing more practical guidance and hands-on examples.

#### Guidance

The guidance by the coordinating centre was attended by 16 stakeholders representing all professions. Among the survey respondents, the guidance was known to 13 out of 17 (76%), and seven of them (54%), mostly ICP (5/7), actually attended this guidance. The guidance was mostly used for exchange of specific information, explanations or practices (5/7). It facilitated the implementation, in particular by providing support when obtaining internal commitment of stakeholders (3/7), assistance in validation of historical data (3/7) and validation of the AS system (3/7).

#### User-group meetings

User-group meetings were attended by 15 persons, representing all professions and hospitals. Among the survey respondents, the user-group meetings were known to 10 of 17 (59%), and attended by 6, mainly ICP (5/6). One orthopaedic surgeon (AS user) and one IPC management staff member (leader at department level) who were not known to the user-group meetings, expressed a desire to have been invited. User-group meetings facilitated implementation and supported in answering questions (4/6), exchanging examples (5/6) and experiences (6/6) with other hospitals and receiving updates from the coordinating centre (5/6). A need was expressed for a forum for questions, and information of other participants, including the phase of implementation and specifications of the EHR system.

The respondents not using elements of the implementation strategy were mostly leader at department level and indicated that the elements were not relevant for their role during the implementation process.

### Barriers and facilitators

**Fig. 3:**
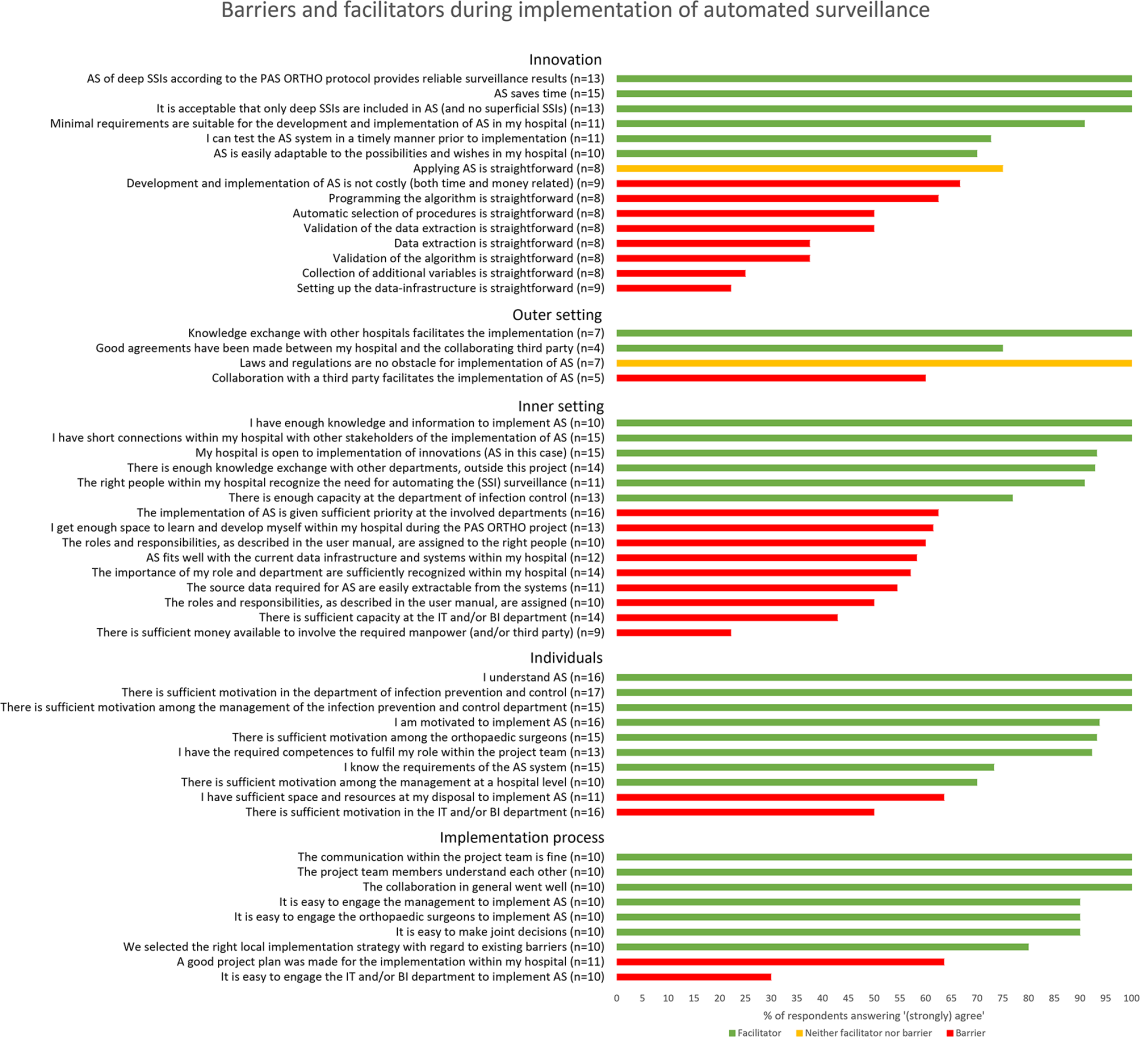
Barriers and facilitators for implementation of automated surveillance. *Barriers and facilitators* [24] based on survey results among stakeholders of the implementation of automated surveillance of surgical site infections after hip or knee arthroplasty. The ‘n=’ behind the propositions indicates the number of total respondents to that specific proposition. For visualisation purposes, the barriers were rewritten to facilitators

In Fig. 3, all barriers and facilitators per domain are shown as included in the survey.

#### Innovation

**Fig. 4:**
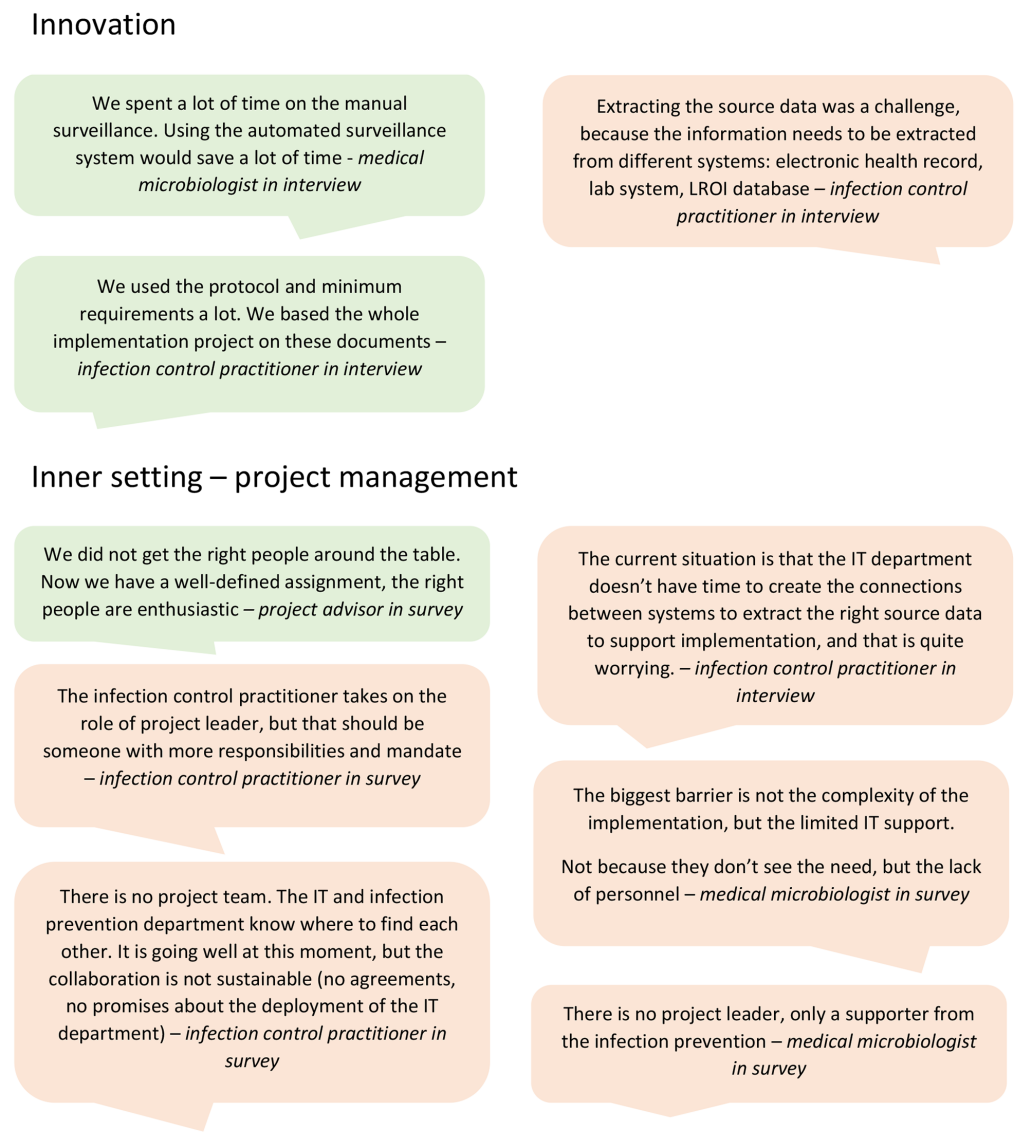
Quotes on barriers and facilitators related to innovation and inner setting (project management)

All respondents believed that AS is timesaving compared to manual surveillance and generates reliable results on incidence of (deep) SSI. Furthermore, the list with minimal requirements, is suitable for the local implementation of AS, according to 91% of the respondents. Barriers related to the complexity of the set-up of the data infrastructure of the AS system (78%), collection of additional variables (75%), data extraction (63%), validation (50–63%), automatic selection of procedures (50%) and programming of the algorithm (38%) (Figs. 3 and 4). All barriers were mainly experienced by ICP who were also involved in the development of AS (developers).

#### Outer setting

Knowledge exchange with other hospitals facilitated the implementation, according to all respondents. Despite reported well-made agreements between hospitals and an external party (e.g. supportive software suppliers) (3/4 respondents), the collaboration with the third party was mentioned as a barrier by 2/5 of the respondents (both ICP) because hospitals are dependent on the pace of work of the third party.

#### Inner setting

All respondents felt they had sufficient knowledge and information to implement AS and that there were short lines of communication between stakeholders of the implementation of AS within the hospitals. However, AS did not fit well with the data infrastructure and systems within hospitals and the source data was not considered easily available from the systems (range 42–46% of the respondents agreed), as experienced by mainly ICP rather than IT specialists. Reasons for these barriers indicated difficulties with connections between systems and the introduction of a new electronic health record system. Furthermore, barriers related to project management were experienced (Figs. 3 and 4). The roles and responsibilities of the implementation of AS were not assigned, and if assigned, not always to the right people (range 40–50% of the respondents agreed), according to mainly ICP fulfilling multiple roles. Some indicated that the role of project leader was not assigned or not to the right person (*n* = 5). Another barrier was the availability of financial resources to involve the required manpower (78%) and capacity at the IT department (58%). The limited IT capacity was not agreed upon by IT specialists.

#### Individuals

The principles and minimal requirements of AS were known by 74–100% of the respondents. The respondents felt that the motivation of the IPC department, orthopaedic surgeons, and hospital – and IPC management was high according to 94–100%. However, 50% (*n* = 8) of the respondents felt that there was lack of motivation at the IT department (Fig. 3). This was mainly experienced by ICP and MM, in the roles of developers, users and/or leaders at department level. The IT specialists (*n* = 2) did not report lack of motivation as a barrier.

#### Implementation process

Survey respondents indicated that the following professions were involved in the decision to start the implementation project: ICP (*n* = 14), medical microbiologist (*n* = 13), infection prevention and control (IPC) management (*n* = 10), orthopaedic surgeon (*n* = 7), IT specialists (*n* = 5), IT management (*n* = 4) and hospital management (*n* = 1). Overall, the collaboration within the local implementation teams was seen as successful (range 90–100%). The engagement of management (hospital - and/or IPC management) and orthopaedic surgeons was reported easy, according 90% of the respondents for both propositions. In contrast, the engagement of the IT department was reported not easy by 70% of the respondents (ICP and MM), thus considered a barrier. In some hospitals there was lack of a structured implementation plan, according to 36% of the respondents (*n* = 4) (Fig. 3).

### Workload for the implementation of AS

**Fig. 5:**
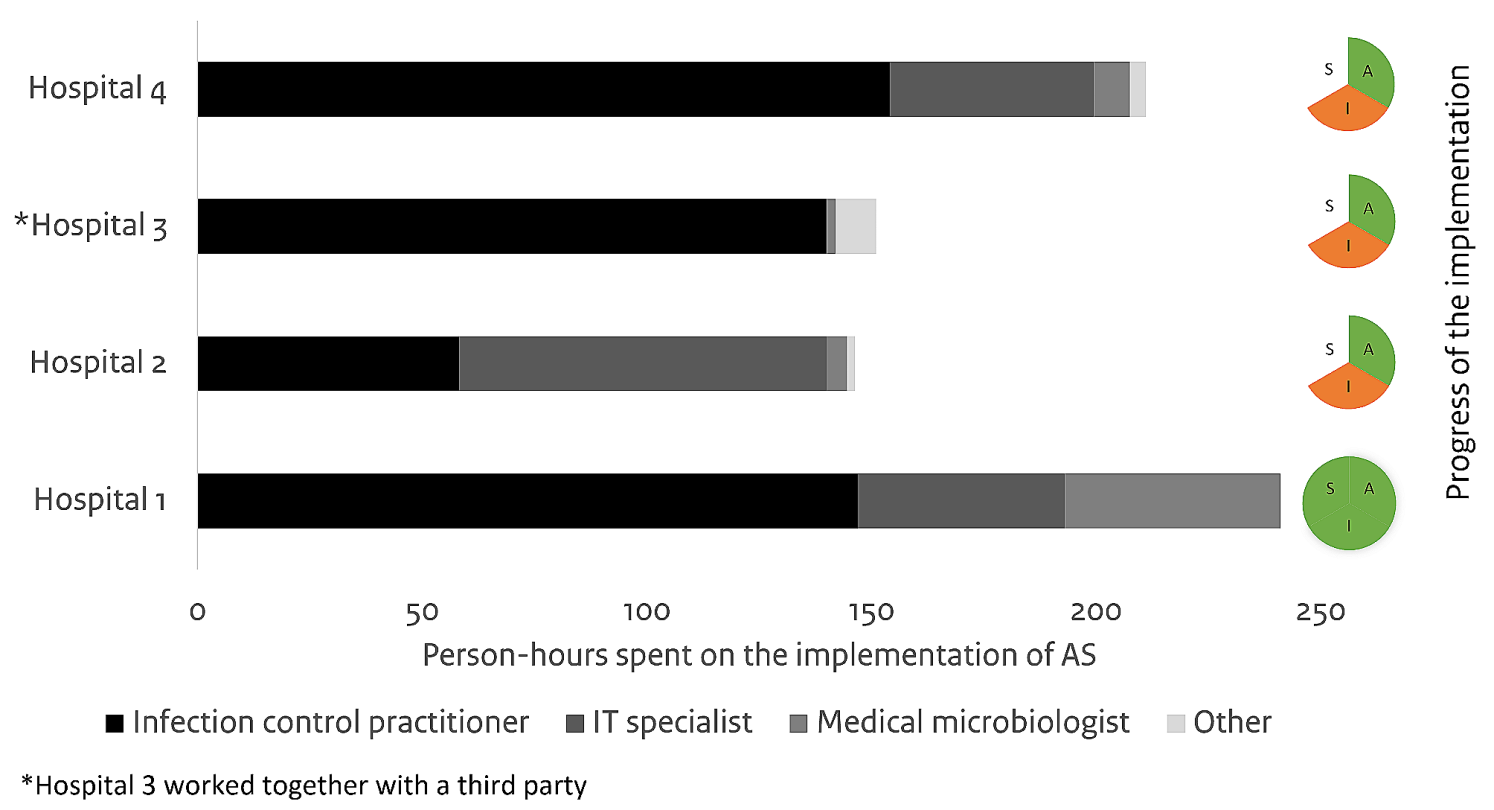
Workload in person-hours per profession per hospital and progress of the implementation. A: adoption phase; I: implementation phase; S: sustainment phase. Green: finished phase; orange; started phase, in progress; white: phase not started

We received time registration of 4/5 hospitals (Fig. 5). The hospital that finished implementation needed 240 person-hours, of which 17% was invested for the validation process. Other hospitals were in various stages within the implementation phase and invested between 145 and 210 person-hours until the end of the study period. One hospital worked together with a third party and had prepared the selection of source data and connections between IT systems in a previous project, hence no person-hours from the IT department were needed.

Overall, predominantly capacity was required from the ICP and IT specialists, with different distributions between hospitals. Although we were not able to link the workload to different roles, the activities of the ICP differed between hospitals. Moreover, the contribution of the medical microbiologists varied between hospitals.

## Discussion

We aimed to evaluate a decentralised multifaceted implementation strategy for semi-automated surveillance of SSI after THA or KA in five Dutch frontrunner hospitals, to provide insight in the effectiveness of an implementation strategy for large-scale implementation of AS. In this pilot study, the implementation strategy appeared suitable for the implementation of AS in hospitals. However, there is room for improvement to enhance the practicality of individual strategy elements.

The effect evaluation revealed that one of the five hospitals managed to finish the complete implementation process during the study period using the multifaceted implementation strategy. Results of the process evaluation provide insight into possible explanations for the difference in time needed for implementation in other hospitals.

Overall, the implementation strategy was positively received and was effective and feasible. The use of a protocol with minimal requirements of the AS system was suitable and in general, active elements (education module, guidance, and user-group meetings), were more effective than passive elements (user manual). The elements of the implementation strategy were used by the intended users, except for managers of the IPC department. During the implementation process, participants perceived the relative advantage of AS and had sufficient knowledge about AS.

This study revealed some persisting barriers to large-scale implementation, including complexity of the local deployment of the AS system, lack of resources, capacity and motivation at the IT department, difficulties with assigning roles and responsibilities and allocating the required capacity, as a result of insufficient project management within the hospitals. The current implementation strategy may not optimally address the identified barriers and improving the implementation strategy based on these barriers may help future implementation efforts. Using the CFIR-ERIC tool [32], useful strategies can be selected to improve the currently used multifaceted implementation strategy. We will illustrate this with examples below when discussing our findings of barriers.

In our study, the complexity of AS, primarily related to the development of the system, was mainly experienced by ICP rather than by IT. Although both professions identify themselves with the role of developers of AS, the background, expertise, knowledge, and tasks of the professions differ. The perceived complexity of AS by ICP may arise from suboptimal project management, or more specifically, the allocation of roles, responsibilities and tasks regarding development of AS. Waltz et al. suggested strategies, including creating a learning collaborative to deal with the complexity of new innovations, i.e. AS [32]. To achieve such a learning collaborative between IT and ICP during the development of AS, it is important to understand each other, speak each other’s language and ask the right questions. Enhancing the utilization of the strategy elements, mainly the education module, by IT would be beneficial for optimizing collaboration between IT and ICP. Furthermore, facilitating knowledge exchange within and between hospitals and providing guidance on allocation of roles, responsibilities and tasks among stakeholders could be included in the guidance and user group meetings. Several other main barriers were related to project management.

Firstly, we found that hospital management or other high-level leaders rarely participated in the project’s adoption phase and the decision to start the implementation project. The involvement of higher management levels from the beginning is necessary to have a clear assignment and allocation of resources to the right stakeholders. Although it has been advised to use a project structure and to involve stakeholders at an early stage, more proactive and practical advices on effective project management, including a clear assignment from management, could be included in the guidance by the coordinating centre to enhance the multifaceted implementation strategy [31, 33, 34].

Secondly, the role of project leader was frequently assigned to ICP, and experienced by the project leaders themselves, as not the person with the right capacities for that role. Furthermore, the IT respondents did not identify themselves as project team member although this was suggested in the user manual. The project leader should be someone with mandate, connections at the right departments and someone who show leadership and ownership. The role of project leader could also be fulfilled by a project manager with expertise in project-based work, instead of a content expert such as ICP. The user manual could give more information about the formation of the project team, and allocation of roles and responsibilities by describing the required competences and tasks of different roles.

Thirdly, we found a lack of resources, mainly in terms of IT capacity which is in line with previous research [30, 31, 35, 36]. Having successful project management could help liberating the required capacity and priority of all stakeholders, including IT, in an early stage of the project. In our study, ICP encountered capacity challenges by IT. However, IT themselves did not report a lack of capacity, which might be explained by low and not representative number of IT respondents, biased towards IT respondents with allocated time for the implementation project. The results regarding the workload for implementation could be useful in allocating the necessary capacity.

### Strengths and limitations

To our knowledge, this is the first evaluation of the implementation of AS among multiple hospitals. Strength of this study is the use of well-known implementation frameworks for the feasibility evaluation and assessment of barriers and facilitators. By including the workload for implementation, hospitals that aim to start with the implementation of AS in the future can use these results to inform involved stakeholders about the expected necessary capacity.

This study also had some limitations. The number of included hospitals and participants was relatively low and the representation of all stakeholders was not optimal. Especially the response rate of the IT and orthopaedic surgeons was limited. However, our aim was to perform a pilot study to improve the implementation strategy is an early stage of the national implementation process, hence limiting the number of possible participants, and the duration of the study period. The limited study period did not allow for measuring actual effectiveness of AS in term of the workload reduction in surveillance performance and maintenance. Furthermore, the frontrunner hospitals evaluated in this study were selected on organisational readiness (e.g. capacity and/or availability of IT department, commitment of stakeholders, access to source data) and likely had more affinity with AS compared to other hospitals, which may limit generalizability. Our results confirmed the importance of these aspects for successful implementation of AS and this led to the recommendation to include support for achieving organizational readiness in the implementation strategy, making the strategy useful and feasible in a broad variety of hospital settings. Moreover, the respondents had the possibility to identify themselves with multiple roles during the implementation; this complicated the distinction of the roles to which the strategy elements were relevant. Another limitation was that due to the anonymous survey, it was not possible to relate answers of the respondents to a specific hospital or phase of implementation. As a result, the outcomes of the effect evaluation could not directly be related to the results of the process evaluation. Furthermore, detailed information about the local situation of hospitals and/or departments in which AS was implemented was lacking, which complicated the interpretability of results [35]. Lastly, the evaluation of the implementation strategy was focused on hospitals, while the coordinating centre was not part of the evaluation. Hence, barriers or facilitators from the central perspective were not part of study.

## Conclusion

This pilot study showed that a decentralised multifaceted implementation strategy was suitable for the implementation of AS in hospitals, but improvements need to be considered. Given that implementing AS is often perceived as complex, especially by ICP, the implementation strategy should facilitate the appropriate allocation of roles, responsibilities, and tasks among stakeholders. A clear assignment and effective project management within the hospitals is crucial in this. Additionally, sufficient knowledge about AS, its requirements and the implementation process should be provided, e.g. by facilitation of the exchange of experiences and best practices between hospitals. Overall, this study suggests that future surveillance networks or centres that aim to implement AS on a large scale, beyond just SSI after THA or KA, could benefit from elements of our implementation strategy, adapted to specific surveillance targets and incorporating the recommendations derived from this study.

### Electronic supplementary material

Below is the link to the electronic supplementary material.

Supplementary Material 1

Supplementary Material 2

Supplementary Material 3

Supplementary Material 4

Supplementary Material 5

## Data Availability

The datasets used and/or analysed during the current study are available from the corresponding author on reasonable request.

## Abbreviations

AS: (semi)Automated surveillance
CFIR: Consolidated framework of implementation research
EHR: Electronic health record
HAI: Healthcare associated infections
ICP: Infection control practitioner
IPC: Infection prevention and control
IQR: Inter quartile range
IT: Information technology or business intelligence
KA: Knee arthroplasty
MM: Medical microbiologists
PRAISE: Providing a roadmap of automated infection surveillance in Europe
RIVM: Dutch national institute of public health and the environment
SSI: Surgical site infection
THA: Total hip arthroplasty
