# Supplementary material for: Evaluation of a multifaceted implementation strategy for semi-automated surveillance of surgical site infections after total hip or knee arthroplasty: a multicentre pilot study in the Netherlands
Source: Antimicrob Resist Infect Control. 2024 Jun 13;13:63. doi: 10.1186/s13756-024-01418-0 (PMC11170835; doi:10.1186/s13756-024-01418-0)
Supplement: Supplementary file 1 — Supplementary Material 1 [file 13756_2024_1418_MOESM1_ESM.pdf]

Supplement 1: Example of questions related to feasibility

The questions in this supplementary file are related to the user manual and are described to get an idea of what was asked in the survey. We asked similar questions for the other elements. Most questions were closed questions with answer options based on results of the interviews, with the possibility to define new answers in an open text field.

Question number	Concept	Question	Remarks
**1**	Implementation	Are you aware of the manual, to be used to support the implementation of AS?	If no: would you have liked to be informed? Why?
**2**	Actual exposure	Have you received the manual?	If no: would you have like to receive the manual? Why?
**3**	Implementation	How did you receive the manual?	
**4**	Actual use	Have you read the manual?	
**5**	Demand	What was the main reason for (not) reading the manual?	
**6**	Practicality	How useful were the following chapters of the manual?	
**7**	Practicality	To what extent did the manual help to achieve the following steps?	
**8**	Acceptability	To what extent are you satisfied with	
**9**	Practicality	When did you use the manual?	
**10**	Acceptability	How could the manual be improved?	
**11**	Acceptability	How likely are you to recommend using the manual to a colleague in the same role as you?	
**12**	Self-reported effectiveness	To what extent has the manual influenced the implementation of AS in your hospital?	
