# Supplementary material for: Evaluation of a multifaceted implementation strategy for semi-automated surveillance of surgical site infections after total hip or knee arthroplasty: a multicentre pilot study in the Netherlands
Source: Antimicrob Resist Infect Control. 2024 Jun 13;13:63. doi: 10.1186/s13756-024-01418-0 (PMC11170835; doi:10.1186/s13756-024-01418-0)
Supplement: Supplementary file 2 — Supplementary Material 2 [file 13756_2024_1418_MOESM2_ESM.pdf]

Supplement 2: Interview guide evaluation implementation semi-automated surveillance

Introduction

1. What is your function in the hospital?
2. For how long have you been working
   - as [profession]
   - in the hospital
   - in the field of infection surveillance

Effect

Adoption/context

1. How did your hospital become involved in this implementation?
   a. How did the decision process go?
   b. Who participated in this decision-making process?
2. What is the extent of your involvement in the implementation of automated surveillance after orthopaedic procedures?
3. What is your experience with decentralized implementation?

   If necessary, ask more specifically:
   a. Degree of freedom/develop and implement automatic surveillance yourself
   b. Data specifications
   c. Minimum program of requirements
   d. Acceptance criteria
   e. Possibility of local adjustments
   f. Support offered (keep it short)

4. What is the current status of the implementation in your hospital?

5. Has a local implementation plan been written?
   a. *If yes:* can you describe it?
      If necessary, ask more specifically:
      1. How detailed?
      2. Is it realistic/feasible?
      3. Who is involved?
      4. What role did the local plan play during the implementation?
   b. *If no:* why not?

Feasibility of strategy elements:

User manual

Actual use

1. To what extent are you familiar with the user manual?

If familiar with the user manual, go to question 2. Otherwise, on to Education.

2. To what extent have you used the user manual?
    a. Why? *(Or why not?)*
    b. How often, and at what times?
    c. For which steps/questions?
        i. Which chapters?
        ii. For local implementation plan?

<u>User manual Chapters</u>

- *Introduction*
- *Background semi-automated surveillance*
- *The project leader and project group*
- *Plan of action (local implementation plan)*
- *Disciplines involved (stakeholders) inside and outside the hospital*
- *Current method of surveillance of SSI*
- *New method of semi-automated surveillance*
- *Barriers and possible solutions*
- *Checklist steps for implementation of semi-automated surveillance*

If you have used the user manual, continue with Experiences. Otherwise, on to Education.

Experiences

What do you think of the user manual?

a. Did you need the user manual during implementation?
b. Does the user manual contain enough information?
    i. How can this be improved?
c. Does the user manual contain the correct level of information?
d. What do you think of the layout of the user manual?
    i. How can this be improved?
e. How accessible do you think the user manual was?
    i. Why is that?
f. What did you think of the way the user manual was presented?
g. Do you think the user manual has facilitated the implementation of automated surveillance?
    i. If not, what needs to change to achieve this goal?

Education module

Our attendance registration shows that you have or have not participated in the education module.

If no:

1. What was your reason for not participating in the education module?

   Move on to user platform

If yes, move on to Experiences.

Experiences

1. What do you think of the education module?
   a. Did you need the education module?
   b. Did the education module contain enough information?
   c. Does the education module contain the right level of information?
   d. What did you think of the structure of the education module?
      i. How can this be improved?
   e. How accessible was the education module?
      i. E.g. way of giving up
   f. Do you think the education module has had an effect on the implementation of the automatic surveillance system?

Guidance by coordinating centre

Actual use

1. To what extent are you familiar with the guidance provided by the coordinating centre?

If you are familiar with the guidance, go to question 2. Otherwise, on to facilitators and barriers.

2. Have you benefited from the guidance provided by the coordinating centre?

If yes:

3. Have you benefited from the guidance provided by the coordinating centre?
   a. Why?
   b. How often, and at what times?
      i. When barriers were experienced?
   c. For which steps/questions?

If no:

   a. Why not?

If the guidance has been used, please go to Experiences. Otherwise, on to facilitators and barriers.

Experiences

1. What do you think of the guidance?
   a. Did you need the guidance?
   b. What did you think of the set-up of the guidance?
      i. How can this be improved?
   c. How accessible was the coordinating centre for the guidance?
      i. Accessibility

    ii. Appointment deadlines

d. Do you think the guidance has had an effect on the implementation of the automatic surveillance system?

Barriers and facilitators

1. Were there any barriers that complicated the implementation?
2. Were there any facilitators that made the implementation a success?

Innovation (automated surveillance)

General

1. Were there any barriers related to the automated surveillance system that stood in the way of implementation? *Example: hard-to-extract data*
    a. In what way do you think these barriers ultimately affected the implementation?
2. Were there any facilitators related to the automatic surveillance system that made the implementation a success? *Example: time saved was motivation*
    a. How do you think these facilitators ultimately impacted implementation?

Based on the answers given, ask further questions, if necessary. on the following points:

Intervention source: Who developed the AS system in/for your hospital? What is your experience with this?

Evidence strength & quality: What types of information are you aware of that shows that automated surveillance works and produces reliable results?

Relative advantage: What benefits do you expect from the implementation of automated surveillance?

Adaptability: To what extent should automated surveillance be adapted to the local situation? Are these options available?

Complexity: How complex is automated surveillance? What makes it complex?

Outer setting (outside the hospital)

General

1. Were there any barriers related to the outer setting, for example collaborations outside the hospital, that complicated implementation?
    a. In what way do you think these barriers ultimately affected the implementation?
2. Were there any facilitators related to the outer setting that made the implementation a success? *Example: knowledge exchange with other hospitals*
    a. How do you think these facilitators ultimately impacted implementation?

Based on the answers given, ask further questions, possibly on the following points:

Cosmopolitanism: To what extent do you collaborate with colleagues outside the hospital in the field of infection surveillance? What knowledge is exchanged?

Inner setting (the hospital, collaboration, working climate)

General

1. Were there barriers related to the inner setting, such as collaborations or culture within the hospital, that stood in the way of implementation?
    a. In what way do you think these barriers ultimately affected the implementation?
2. What are facilitators for the implementation that had to do with the inner setting, i.e. the hospital?
    a. How do you think these facilitators ultimately impacted implementation?

Based on the answers given, ask further questions, if necessary. on the following points:

Infection prevention (IP) and IT collaboration: How did the collaboration between IP and IT go during this implementation project?

Implementation climate: How is the implementation of automated surveillance generally received in your hospital?

Tension for change: Is there a strong need for automatic surveillance in the hospital? Why?

Compatibility: How well does automatic surveillance fit within the existing work processes within the hospital?

Relative priority: What high-priority activities or initiatives are already taking place in the hospital, linked to automated surveillance? (e.g. implementation of new EPD)

Goals and feedback: Have you, your department, or your hospital set goals related to the implementation of automated surveillance? If so, can you describe them?

Learning climate: To what extent do you feel that you can try new things to improve the work processes in your hospital?

Readiness for implementation

Leadership engagement: What level of approval or support for this implementation is there from the hospital management?

Available resources: Do you expect to have sufficient resources to implement automatic surveillance? Which one? Which ones are missing?

Individual characteristics

General

1. Were there any barriers related to your own ideas or skills that complicated implementation?
    a. In what way do you think these barriers ultimately affected the implementation?

2. Were there any facilitators related to your own ideas or skills that made the implementation a success? *Example: a lot of confidence in AS so extra motivation*
    a. How do you think these facilitators ultimately impacted implementation?

Based on the answers given, ask further questions, if necessary. on the following points:

Knowledge & beliefs about the intervention: What do you think about automatic surveillance being used in your hospital?

Self-efficacy: How confident are you that you are able to successfully implement automated surveillance? And to use?

Individual stage of change: How well prepared are you to move to automated surveillance?

a. Knowledge phase
b. Decision phase
c. Implementation phase
d. Mounting phase

Other personal attributes/Needs: How well do you think the automated surveillance system meets your needs? In what ways?

Process

General

1. Have you run into barriers related to the implementation process?
    a. In what way do you think these barriers ultimately affected the implementation? *Example: IT people weren't as involved*
2. What are the facilitators to the implementation that had to do with the implementation process? *Example: involving all stakeholders in the process at an early stage*
    a. How do you think these facilitators ultimately impacted implementation?

Based on the answers given, ask further questions, possibly on the following points:

Engaging

Opinion leaders: Who are the key people who need to be involved in the implementation?

Formally appointed internal implementation leaders: Who is in charge during the implementation of AS?

External Change Agents: Are there people from outside your hospital involved in the implementation of AS? Who?

Key stakeholders: What steps have been taken to get individuals to agree to the implementation of AS?
