# Supplementary material for: Evaluation of a multifaceted implementation strategy for semi-automated surveillance of surgical site infections after total hip or knee arthroplasty: a multicentre pilot study in the Netherlands
Source: Antimicrob Resist Infect Control. 2024 Jun 13;13:63. doi: 10.1186/s13756-024-01418-0 (PMC11170835; doi:10.1186/s13756-024-01418-0)
Supplement: Supplementary file 3 — Supplementary Material 3 [file 13756_2024_1418_MOESM3_ESM.pdf]

Guideline specifying and reporting of implementation strategies

Proctor, E.K., Powell, B.J. & McMillen, J.C. Implementation strategies: recommendations for specifying and reporting. *Implementation Sci* **8**, 139 (2013). https://doi.org/10.1186/1748-5908-8-139

Prerequisites	Requirements	Checklist
**1) Name it**	Name the strategy, preferably using language that is consistent with existing	Methods – multifaceted implementation strategy Table 1A: columns represent strategy elements
**2) Define it**	Define the implementation strategy and any discrete components operationally	Methods – multifaceted implementation strategy Table 1A : columns represent elements and row 'content'
**3) Specify it**		
**a) The actor**	Identify who enacts the strategy (e.g., administrators, payers, providers, patients/consumers, advocates, etc.).	Table 1A : row ' developed by'
**b) The action**	Use active verb statements to specify the specific actions, steps, or processes that need to be enacted.	Table 1A : row 'content'
**c) Action target**	Specify targets according to conceptual models of implementation<br><br>Identify unit of analysis for measuring implementation outcomes	Table 1A : row 'intended users'
**d) Temporality**	Specify when the strategy is used	Table 1A : row 'timing roll-out'
**e) Dose**	Specify dosage of implementation strategy	N.A. for this study
**f) Implementation outcome affected**	Identify and measure the implementation outcome(s) likely to be affected by each strategy	Table 1A : row goal
**g) Justification**	Provide empirical, theoretical, or pragmatic justification for the choice of implementation strategies.	Methods – multifaceted implementation strategy
